# Systematic review of the use of debates in health professions education – does it work?

**DOI:** 10.3205/zma001245

**Published:** 2019-08-15

**Authors:** Ren Xuan Ang, Qian Hui Chew, Min Yi Sum, Somnath Sengupta, Kang Sim

**Affiliations:** 1 Tan Tock Seng Hospital, Psychological Medicine, Singapore; 2 Woodbridge Hospital, Institute of Mental Health, Research Division, Singapore; 3 Woodbridge Hospital, Institute of Mental Health, West Region, Singapore

**Keywords:** education, health professions, debate

## Abstract

**Background:** It is unavoidable for learners undergoing health professions training to encounter different complex clinical scenarios related to diagnoses, treatment and ethical dilemmas. The lack of standard answers to such clinical challenges can cause uncertainty in the process of teaching, learning and assessment as learners grapple with the need to critically appraise the specific context, past practices and extant literature to arrive at a thoughtful decision. In this aspect, debate can be a useful pedagogical tool to consider multiple, different perspectives regarding these issues. As the use of debate within the health professions education has not been reviewed, we conducted a systematic review of the current literature on the adoption of debate as a pedagogical tool to clarify the specific context of use as well as its effectiveness in the learning of skills and content within the health professions education.

**Methods: **A systematic review was conducted on relevant published literature in English within journal databases until September 2018 that employed debate as a pedagogical tool within health professions education.

**Results: **Out of 626 screened articles, 12 studies were finally included based on inclusion/exclusion criteria. The 12 studies (9 undergraduate, 3 postgraduate) covered a diverse range of disciplines and debate in health professions education was adopted for acquisition of skills (such as critical thinking, communication skills, teamwork), or learning of specific topics (such as patient safety, ethical issues, teaching of new topics) as well as to examine evidence based practice. In the majority of studies (10 of 12 studies, 83.3%), debate has been deemed by the learners to be effective in facilitating the learning of new content and skills such as communication and critical thinking, which are related to processes aligned with adult learning, motivational, reflective and social learning theories.

**Conclusion: **Overall, sparse studies to date with relatively low risk of bias found debate to be effective in enabling the learning of skills and content within health professions education. Future studies may want to incorporate more objective measures of the learning outcomes of learners following the adoption of debate as a teaching tool as well as to examine the combinatorial use of debate with other pedagogical tools and their longitudinal impact on learners and learning.

## Zusammenfassung

**Hintergrund: **Für Lernende während der ärztlichen Ausbildung ist es unvermeidbar, verschiedenen komplexen klinischen Szenarien hinsichtlich Diagnose, Behandlung und ethischer Dilemmata zu begegnen. Der Mangel an Standardantworten auf solche klinischen Herausforderungen kann zu Unsicherheit im Prozess des Lehrens, Lernens und der Einschätzung führen, da die Lernenden sich mit dem Bedürfnis nach kritischer Bewertung eines spezifischen Kontextes, vergangenen Praktiken und vorhandener Literatur auseinandersetzen müssen, um zu einer durchdachten Entscheidung zu gelangen. In dieser Hinsicht kann die Debatte ein nützliches pädagogisches Werkzeug sein, um verschiedenartige, multiple Perspektiven bezüglich dieser Probleme zu berücksichtigen. Da der Nutzen von Debatten innerhalb der ärztlichen Ausbildung noch nicht überprüft worden ist, haben wir einen systematischen Überblick über die aktuelle Literatur zur Einbeziehung von Debatten als pädagogisches Werkzeug vorgenommen, um deren spezifischen Nutzungskontext sowie Effektivität beim Erlernen von Fähigkeiten und Inhalten innerhalb der ärztlichen Ausbildung zu klären.

**Methoden: **Es wurde eine systematische Überprüfung der relevanten, bis September 2018 veröffentlichten englischsprachigen Literatur in Zeitschriftendatenbanken durchgeführt, die sich mit Debatten als pädagogischem Werkzeug innerhalb der Ausbildung von Gesundheitsberufen befassten.

**Ergebnisse: **Von 626 angezeigten Artikeln wurden schließlich 12 Studien basierend auf Inklusions-/Exklusionskriterien einbezogen. Diese 12 Studien (9 universitäre, 3 postgraduierte) deckten ganz verschiedene Bereiche und Disziplinen ab, und die Debatte in der Ausbildung von Gesundheitsberufen wurde zum Erwerb von Fähigkeiten (wie z. B. kritisches Denken, kommunikative Fähigkeiten, Teamwork) oder zum Erlernen spezifischer Themen (wie z. B. Patientensicherheit, ethische Probleme, Unterrichten neuer Themenbereiche) sowie zur Überprüfung Evidenz-basierter Praxis genutzt. Bei der Mehrheit der Studien (10 von 12 Studien, 83,3%) wurden Debatten seitens der Lernenden als effektiv bei der Erleichterung des Erlernens neuer Inhalte und Fähigkeiten, wie z. B. bei der Kommunikation und dem kritischen Denken, eingeschätzt, die verbunden sind mit Prozessen, die am Lernen im Erwachsenenalter, an Motivations-, Reflexions- und sozialen Lerntheorien ausgerichtet sind.

**Schlussfolgerung:** Insgesamt schätzten die bislang nur spärlich vorhandenen Studien mit einem relativ niedrigen Risiko der systematischen Verzerrung Debatten als effektiv bei der Ermöglichung des Erlernens von Fähigkeiten und Inhalten innerhalb der Ausbildung von Gesundheitsberufen ein. Zukünftige Studien sollten eventuell objektivere Maßstäbe bzgl. der Lernergebnisse nach Einbeziehung von Debatten als Lehrwerkzeug anlegen, sowie die kombinierte Nutzung von Debatten zusammen mit anderen pädagogischen Werkzeugen und deren langfristigen Einfluss auf Lernende und Lernen untersuchen.

## 1. Introduction

In the process of health professions training and practice, learners will inevitably encounter complexity and controversies related to diagnoses, treatment, ethical conundrums and policy issues [1], [2]. Teaching, learning and assessment in such contexts can be fraught with uncertainty as there are no standard answers and responses require multi-faceted understanding, critical and comprehensive appraisal of the specific situation and context. In this aspect, debate can be an appropriate pedagogical tool to expand the scope of learning to consider multiple perspectives which would be useful when tackling these conflicts and controversies [3]. 

It was evident even from Ancient Greek history that debate hones critical thinking for formulation of arguments and effective communication skills so as to persuade the listeners towards one’s point of view [4]. The process of debate involves consideration of multiple viewpoints, reflection, internalization and then arriving at a reasoned opinion about an issue, and can occur in both individual and group settings [5]. For individuals, an internal mental process takes place before a specific decision is made whilst for a group, this often involves active deliberation and argumentation over the facts and differing facets of the issues [5]. Debates as a teaching method has been previously employed in a variety of disciplines, ranging from political science [6], mathematics and statistics [7], argumentative writing [8], to healthcare [9], [10], [11]. Though debates are often conducted in the traditional classroom setting, it can be effective even if an online format is adopted [12]. The strengths of using debate as a pedagogical tool include greater engagement of learners, encouragement of deeper exploration and consideration of issues, and inculcation of communication skills to articulate relevant points clearly and concisely [13], [14], [15]. Some limitations include the requirement for a specific controversial theme and not just a topic requiring factual recall, time consuming preparation for learner and faculty, and that there may be other more time-efficient methods with comparable learning outcomes such as team-based learning [16], [17].

How debate facilitates learning may be linked to principles of learning related to adult learning, constructivism and deliberate practice models. The adult learning theory [18] emphasizes that adults are self-directed and expect to take responsibility for their learning. Hence, learning takes place best when learners are actively involved, when it is problem-centered, and when the learner sees the immediate value of the topic. In this aspect, the preparation for the debate tackles a specific controversial theme (problem) which allows for experiential learning as the learner is actively engaged (as a member of the proposition or opposition team) in the process of looking at different perspectives of the motion for the debate. Constructivist theory [19] posits that learning is an active process whereby the learner constructs new ideas or concepts from current or past knowledge which is pertinent for the learner-debater as he makes sense of the amassed information that is relevant to the topic of interest. Following better engagement, deliberate practice [20], which involves constant, repeated practice of the material to be presented using tools of reflection and feedback, can further enhance the learning with debate. 

Despite various potential benefits of debates as a pedagogical tool, its adoption, context of use, and effectiveness within the health professions education have not been fully examined. Hence, we sought to perform a systematic review of the current literature on the adoption of debate as a pedagogical tool in order to clarify the specific contexts of use (such as undergraduate vs postgraduate, discipline), as well as its effectiveness within health professions education as defined by improvement in learning of skills or content via feedback ratings. 

## 2. Methods

We designed and reported this review based on the principles of the Preferred Reporting items for Systematic Reviews and Meta-analyses (PRISMA) statement [21]. We searched several large journal digital databases, namely ScienceDirect, Scopus, EMBASE, and PubMed/Medline from inception until September 2018 for relevant studies involving the pedagogical use of debates in health professions education. We used multiple medical journal search engines in order to ensure comprehensive identification of all relevant articles. We used combinations of the following keywords, namely “debate”, “health professions education” and “medical education”, to constitute three search strings, namely, “debates” AND “medical education”, “debates” AND “health professions education” as well as combination of all the three terms. The reference lists of retrieved articles were also examined to identify other relevant articles. Inclusion criteria were:

1. empirical studies involving an evaluation of debates as a pedagogical tool,
2. conducted within the context of health professions either at undergraduate or postgraduate level and
3. availability in the English language.

Exclusion criteria were:

1. studies involving debates as pedagogical tool outside of health professions or medical education and
2. not reported in English. Letters to the editor and conference abstracts were excluded.

All researchers screened the abstracts of identified potentially useful reports to ascertain whether they met the inclusion criteria and then reviewed full reports of promising studies, and screened their bibliographies for additional references. Disagreements regarding the inclusion or exclusion of studies, and captured details of studies were resolved via team consensus meetings in which the reports were reviewed by all authors. For each individual study, we extracted variables including the stated aims, characteristics of the learners, details of pedagogy, measures used if any, and salient findings of the studies as well as scoring of likely attrition or reporting bias which was modified from Cochrane Collaboration’s tool for assessing bias in randomized controlled trials [22]. Attrition bias is bias arising from incomplete outcome data and reporting bias is bias arising from selective reporting of outcomes [22]. The preceding data were organized in digitalized spreadsheets and then summarized in a table to guide preparation of critical assessments included in this manuscript and for independent consideration by readers. 

## 3. Results

**Figure 1:**
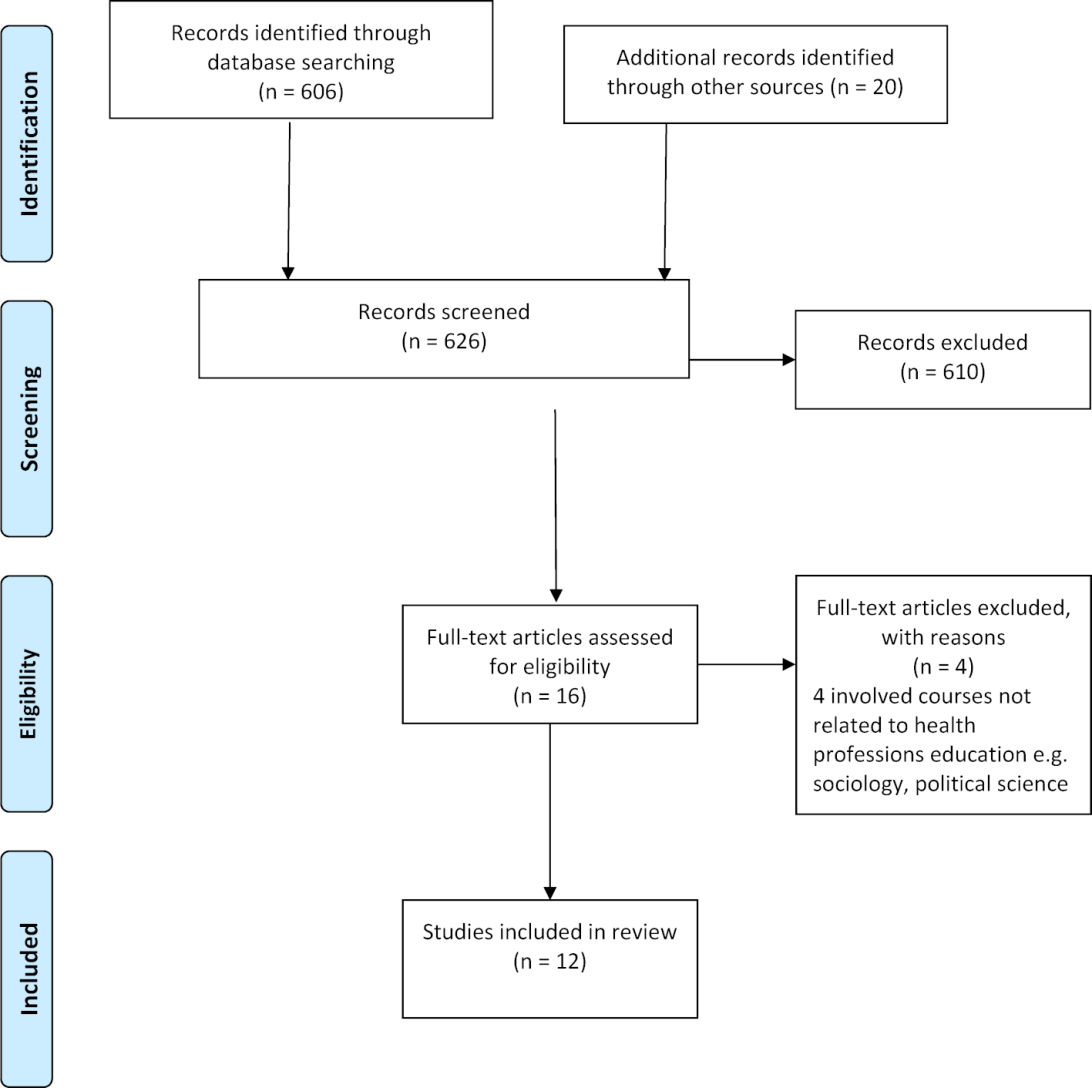
PRISMA flowchart of the literature search and study selection process in a review of the use of debate in health professions education, published up till September 2018.

Attachment 1 summarises the main characteristics of the studies within this review including aims of the study, learner features, details of pedagogy, measures used as well as significant findings and risk of bias. The PRISMA flowchart for this review is shown in figure 1 (Fig. 1). Altogether, 16 reports were identified using our search criteria. Four references were removed as they involved non-health professions, thus leaving 12 studies for inclusion in this review. 

### 3.1. Specific contexts when debate is used as a pedagogical tool

In terms of the level of health professions training, 9 studies (75%) pertained to the use of debates in undergraduate education and 3 (25%) in postgraduate education. Debate was adopted as a pedagogical tool across a wide range of disciplines. Out of the 9 studies within undergraduate education, 3 involved dental students, 3 focused on pharmacy students and the remaining 3 involved medical students, occupational therapy and nursing students respectively. The 3 studies within postgraduate education were related to residents in emergency medicine, paediatric surgery and physical medicine and rehabilitation respectively. Debates were used for three key purposes, the first being development of key skills within health professions education such as effective communication, critical thinking skills, and higher order cognitive skills [4], [9], [23]. Second, five studies used debates as a tool to introduce and teach important healthcare topics such as patient safety [10], health economics [11], medical informatics to medical students [24], healthcare reform policies [25] and microbiology to nursing students [26]. Third, debates were used to teach evidence-based management using case-based scenarios [27]. Debates were conducted in the form of classroom presentations of pre-assigned topics by the faculty [4], with pre-and post-debate assessments using quizzes and surveys [9], [10], [11], [13] or judged by the audience vote [27]. Another study also used online discussions among paired student groups and the topics and the sides were randomly assigned [23].

#### 3.2. Effectiveness and evaluation of debate in the learning of skills and content 

Overall, the majority of the studies (10 of 12 studies, 83.3%) were assessed to be at low risk of attrition or reporting bias, and they reported that debate was effective as a pedagogical tool within different unique learning contexts in enhancing the learning of skills or content. This was evaluated by some studies using measures such as a Likert scale [10] or additional focus group discussions [24].

How does debate facilitate the learning of skills and content? First, debate encourages evidence-based learning [4]. Preparation of debate requires participants to consider thoroughly the evidence in order to present and argue persuasively for their case. One of the studies [10] that used a Likert scale to measure the impact of debate reported that 71% of residents agreed or strongly agreed that their individual performance on a specific topic related to patient safety and quality improvement had improved by critically appraising the scientific literature and applying evidence-based medicine. In the same study [10], approximately a third of the residents led a debate. Of those that led, 100% agreed or strongly agreed that they could propose an evidence-based approach to that specific topic.

Second, it fosters the learning of research skills. The process of preparing for debates requires participants to undertake extensive research in order to formulate convincing arguments and substantiate it with evidence. Some of the studies reviewed showed a significant improvement in the participants’ research skills pre and post-debate [4], [9], [28]. In a study involving occupational therapy students [13], 87% of them responded that they would not have researched the issues on their own if not for the debate. Third, debate preparation promotes content reinforcement by encouraging participants to crystallize and integrate relevant information from various sources, and subsequently craft them into logical arguments [4], [29]. Fourth, the process of debate also develops communication and public speaking skills as participants were required to discuss and communicate their ideas within the small group [28]. Fifth, debate promotes active listening [4] amongst participants, both during preparation of debate arguments as well as during the debate itself. Debating, hearing and discussing the debate content serves to reinforce learners’ understanding of text material, and audience participation allowed for extended active discussion of the debate topic pertaining to healthcare [13], thus enhancing the depth of content learned as well. 

Sixth, the use of debates facilitates critical thinking and evaluation in debate participants [9], [28]. Constructing arguments encourages participants to examine issues from different vantage points, thereby reaching a more balanced conclusion through the repeated process of questioning and self-evaluation [4], [9], [10]. Seventh, the use of debates strengthens teamwork [9]. This is a particularly important skill in the health professions as multidisciplinary practices are common and require effective communication within and amongst teams. Eighth, students perceived the learning process to be more interesting with the use of debates as compared to a didactic teaching style [10], [11], and this can in turn motivate pursuit of content knowledge both within and outside of the classroom. In a study conducted among paediatric surgery residents [27], it was reported that 87% of the audience found it a new and enjoyable style of learning and 75% wanted more of such sessions. In addition, debate was found to be an effective modality in knowledge acquisition with feedback through quizzes before and after the debate, and this was true for not just for debate participants but also the audience [11], [24], [25], [27], [29].

## 4. Discussion

Despite relatively sparse data, this study revealed that debate has been adopted as a teaching tool across several healthcare disciplines especially at the undergraduate level. It was generally found to be effective for facilitating learning within health professions education. Debate was used for different purposes such as to develop skills (e.g. critical thinking, communication, teamwork), address specific issues (e.g. patient safety, healthcare policies), and examine evidence-based practice. We will first attempt to discuss the rationale for its effectiveness using various theories and models of learning, followed by possible caveats regarding its use, implications for educators, and limitations related to our review.

Adult learning theory [18] considers that learning is enhanced when a learner sees the need and immediate relevance of the learned content, when there is a clear problem focus, and when learning is experiential in nature. During debate preparation, the learner identifies the issue of focus, and sees the need to research the topic in order to appreciate the different perspectives related to the evidence available. This was observed in a study involving a group of emergency medicine residents who found that the process improved their ability to critically appraise the scientific literature about patient safety issues [10]. In addition, the learner applies knowledge acquired during research to prepare the logical argument which is crucial in a debate; hence learners have found debate helpful in inculcating research skills [13]. The audience can also better acquire knowledge as they are engaged in the cut and thrust of the debate experientially [27]. 

Consistent with the self-determination theory [19], debate can also enhance engagement and motivation, and the process of preparation fosters a sense of autonomy, competency and belonging within the team. In one report where paediatric surgery trainees presented opposing views of management of a case and the outcome was judged by the audience, 87% of the students found it a new and enjoyable style of learning while 75% wanted more of such sessions [27]. Once engaged in learning, the learner constructs meaning within the examined material, achieves understanding, and assigns significance to what has been learned. This is supported by evidence indicating that learners appreciated how debate preparation helped to develop critical thinking skills through distillation and integration of summated information [4], [9], [28]. Furthermore, the need for active listening for alternative viewpoints [4] and reflective learning [30], [31] during the debate preparation serve as tools for deliberate practice which further refines the presentation and rebuttal during the actual debate. These aspects were observed in the use of debates for developing competencies in dental undergraduates [4] and even disciplines outside of health professions education whereby listening to, and reflective discussion of the debate content increased one’s understanding of the topic of interest [16]. 

In addition, consistent with social learning theory [32], learning as a social process [28] occurs when there is acquisition and subsequent application of knowledge in a debate setting. This was observed by Lampkin et al (2015) [9] when debate was used to develop skills including teamwork and respondents reported significant improvement in this area following the debate series. However, it is also noted that team members must encompass trust, respect and honesty for each other for debate to be a useful tool [33]. 

In summary, through the use of debates as a teaching tool, learners are better engaged and motivated to learn (self-determination theory). The significant aspects of debate preparation include the presence of a debate theme (problem focus) which allows for appreciation of the need for reading around the topic (need to know, readiness to learn), conducting research (experiential learning), and the understanding that these efforts would aid the collation of debate content (immediate relevance). In the process, there is distillation and integration of knowledge acquired to construct logical arguments (constructivism). The refinement and persuasiveness of the arguments require constant practice (deliberate practice), with active listening, reflective learning and feedback as important tools. Throughout this journey, there is continual interaction with different team members, which fosters co-operation and learning within a learning community (social learning). In Anderson’s modifications of Bloom’s taxonomy [34] with learning outcomes ranging from remembering, understanding, applying, analyzing to evaluating and creating, debate allows for learners to traverse and progress across these different learning outcomes with the aim of ultimately being able to create arguments from critical reading and discussion about the theme. This was in agreement with Omelicheva et al.’s [35] findings that lectures were effective in promoting memorization of basic subject knowledge, whilst debates were useful in addressing controversial topics, developing comprehension of complex concepts, and critical evaluation, analytical, and application skills. 

There are several caveats on the employment of debate for health professions education. Effectiveness of debate as a teaching and learning tool is largely dependent on the preparation done by learners, and yields greater educational benefit when extensive preparation and research is done [13], [23]. Classes also need to be kept small in order to maximize effectiveness and to encourage audience participation [23]. Hence, it might not be the most ideal teaching tool for large cohorts. In addition, the ability to participate effectively in debates is not always intuitive for all learners. Familiarity with the style of debate, being comfortable with public speaking and arguing for and against a motion are skills that might need training and exposure [13]. Hence, those that are uncomfortable with this pedagogical tool might not be able to maximize their learning. The process and technique of constructing sound, evidence-based debate arguments are also important in determining the effectiveness of debates. Without these skills, debates tend to become summaries of current literature rather than robust evaluations [16].

Bearing in mind the benefits and caveats mentioned above, there are several points to note for health professions educators who may want to adopt debate in their clinical teaching. First, educators may consider incorporating debate in their teaching especially if the goals pertain to acquisition of skills, addressing controversial topics, or examination of evidence-based practice. Second, debate-based teaching sessions within the curriculum need to be identified early as adequate preparation by the educator and learners contributes to teaching effectiveness. Third, the educator needs to be cognizant that debate is more suited for smaller class sizes which allow for active participation of all learners. Fourth, learners should be regarded as adult learners and given the space to conduct their own research and discussion about the topic of interest. Fifth, options for different debate formats can be considered as some learners may not be comfortable with debating in person but may have no qualms about active participation on an online platform [12]. 

There are several limitations related to this review. First, we limited it to published studies in English. Second, there was a paucity of studies which compared debate with another common pedagogical modality such as lectures within health professions education which would have proffered greater insight into its additional value in learning and teaching. Third, whilst there were evaluations of the debate itself in included studies, there was often no objective measure of the skills acquired (e.g. communication, critical thinking) or understanding of the content delivered which would complement subjective feedback given by the learners. Fourth, there is no study on the sustained use of debate in teaching or long term learning outcomes following the adoption of debate as a teaching tool. Fifth, more studies of debates adopted in postgraduate education are warranted.

## 5. Conclusion

In conclusion, within the limited number of studies included, the majority has studied debate as a pedagogical tool predominantly at the undergraduate level and focused on acquisition of skills or learning of specific topics. Debate has been deemed by the learners to be effective in the learning of new content and skills such as communication, critical thinking, teamwork which may be related to processes aligned with adult learning, motivational, reflective and social learning theories. Future studies may want to incorporate more objective measures of learning outcomes following the adoption of debate as a teaching tool, as well as to examine the combinatorial use of debate with other pedagogical tools and their longitudinal impact on learners and learning. 

## Author contributions

KS and RXA planned and designed the study; RXA, QHC, MYS and KS were involved in the acquisition of relevant literature; all authors were involved in the interpretation of the data; and all authors contributed to and have approved the final manuscript.

## Competing interests

The authors declare that they have no competing interests. 

## Supplementary Material

Main Characteristics of the Included Studies
