# Supplementary material for: Systematic review of the use of debates in health professions education – does it work?
Source: GMS J Med Educ. 2019 Aug 15;36(4):Doc37. doi: 10.3205/zma001245 (PMC6737264; doi:10.3205/zma001245)
Supplement: Main Characteristics of the Included Studies [file JME-36-4-37-s-001.pdf]

Authors	Aim	Learner features	Details of Pedagogy	Measures used	Salient Findings
Darby 2007	To teach topics with controversial issues in the US dental healthcare system and develop competencies in communication and critical thinking	Undergraduate dental students at Old Dominion University (USA)	Pre-debate – 8 to 10 students in one team to debate on an issue<br><br>Debate – 10 to 12 minute presentation/argument based on evidence-based material<br><br>Debate structure – constructive argument, rebuttal and class interaction phase, each debater needs to come up with 1 good test question that could be used in the final exams. There was evaluation of student's performance both in terms of content and delivery.	NA	Debate encouraged researching current issues, actively listening to various perspectives, integration of information based on evidence.<br><br>**Effective: Yes**<br>**Attrition/Reporting Bias: Moderate**
Hanna et al 2014	To address ethical issues	Undergraduate 2nd year pharmacy students at Queen's University Belfast (UK)	Debates occurred over course of 2 workshops. Guidelines given to students and facilitators 4 months before debates.<br><br>Assessment – each debate team received an overall score which comprised of both peer and staff member contributions. Assessment forms also had section for written feedback.	8-question evaluation questionnaire	70% strongly agreed that debate was a useful learning method, 84% students better developed their research skills, 81% communication skills, 81% teamwork skills, 53%, critical-thinking skills; and 50%, rebuttal skills.<br><br>**Effective: Yes**<br>**Attrition/Reporting Bias: Low**
Khan et al 2012	To assess the perceptions about debate as a learning activity for health economics	Undergraduate third year dental students at International Medical University, Kuala Lumpur (Malaysia)	Parliamentary style of debate chosen, lead debater presented resolution, defined essential terms, presented affirmative case. Members of each team supported their team's arguments and rebutted each other. Judges provided feedback after debate followed by open discussion by the house.<br>Debate judged by 3 senior academicians from 3 disciplines	4-item questionnaire addressing the students' perceptions about the use of debate in learning the topic (health economics in dentistry)	Students felt that their interest in debate, knowledge of the topic, and reinforcement of the previous knowledge had improved following participation in the debate. Students who rated "Very High" interest in the debate topic increased by 32.9%. Students who rated "Very High" knowledge of the debate topic also increased by 32.9%. 30.2% more students also felt that the debate helped them to organize thoughts on the topic

			(Dentistry, Medical Education and English Language)		greatly after participation. 24.6% more students also felt that knowledge regarding the topic was reinforced after participation.<br><br>**Effective: Yes**<br>**Attrition/Reporting Bias: Low**
Lampkin et al 2015	To evaluate the usefulness of formal debates as a tool for developing competency in skills such as critical thinking, communication, teamwork	Undergraduate first year pharmacy students at D'Youville College School of Pharmacy (US)	Structure - 5 debates, each focused on controversial self-care topic<br><br>Report – each team required to submit a report about their topic on the day before their debate<br><br>Post-debate – self and peer evaluated evaluation rubrics Assessments comprised of pre-debate and post-debate quizzes, pre-course and post-course surveys	Predebate and postdebate quizzes (used to assess students' understanding of the material before and after a debate);<br><br>Precourse and postcourse surveys (self-assess perceptions of learning subject material and developing critical-thinking, oral and written communication, public-speaking, research methods, and teamwork skills during the debate series)	Quiz results – Average grade on post-debate quizzes showed a significant improvement in understanding up to 36%.<br><br>Significant self- reported student feedback on improvement (from 52% to 71%) for critical thinking, public speaking, research methods, and teamwork<br><br>**Effective: Yes**<br>**Attrition/Reporting Bias: Low**
Lieberman et al 2000	To evaluate use of structured student debates for promoting the development of several cognitive and informatics-related skills	Undergraduate second year medical students at University of Texas Medical Branch (US)	Students received assigned topics at beginning of course, each student participated in 1 debate. A faculty moderator kept the session on schedule, participated in the question-and-answer portion, and evaluated the students' performances.<br><br>Assessment: individual - presentation skills, contributions to the rebuttal and question-and-answer segments, and professionalism. Teams - accuracy of information and appropriateness of conclusions, written summaries and	Post-debate survey (*n* = 174);<br><br>Faculty moderators (*n* = 17) were surveyed to obtain their impressions of the students' skills and the educational value of the debates;<br><br>Two focus groups of randomly selected students (*n* = 4 per group) met with facilitators midway through the course to discuss the debates and other course aspects	67% of students indicated skills acquired through debate would be 'important' or 'very important' in their careers.<br><br>Students' feedback showed significant improvement for all skills, with mean ratings of post-debate skills at 'moderately competent' levels.<br><br>**Effective: Yes**<br>**Attrition/Reporting Bias: Low**

			references turned in at the debate.		
Lin et al 2007	To facilitate group process, introduce controversial issues related to the US healthcare system, improve critical thinking and communication skills.	Undergraduate pharmacy students at the University of Illinois at Chicago (US)	Online debate – word limit enforced for each online 'speech'<br>Each student received 2 articles to read, one from each side of the debate issue. Deadlines given to post arguments online. Hard copies of posted arguments printed for grading purposes.<br><br>Points – deducted when arguments exceed word limit, no proper citation. Awarded to winning teams as judged by course coordinator and graduate teaching assistants.<br><br>Assessment – each debate group assessed by head judge and 2 other judges, head judge would write summary critique.<br><br>Debriefing sessions held weekly among teaching coordinators	Open-ended post-debate evaluation form	48.1% indicated that learning more about debate topic was most beneficial, 26.5% indicated working as team member and considering alternative positions were most beneficial<br><br>**Effective: Yes**<br>**Attrition/Reporting Bias: Low**
Mamtami et al 2015	To develop a novel curriculum for emergency medicine (EM) residency training program specially addressing patient safety, systems- based management, and practice-based performance improvement	EM residents at University of Pennsylvania Perelman School of Medicine (US)	Moderator introduces topic, poses questions to debaters and audience.<br><br>Teams have 15 mins to present opposing views for the diagnosis, treatment, and/or disposition of commonly encountered ED presentations using the best available evidence. Audience has 15 mins to discuss opposing viewpoints and determine if consensus can be reached.	The goals for the debate participants include describing patient safety concepts (sub-competency 16, level 3), and the ability to critically appraise literature and apply evidence-based medicine (sub-competency 19, level 3).	Residents survey:<br>71% of residents agreed/strongly agreed that they have improved in critical appraisal of scientific literature. 68% agreed/ strongly agreed they could describe patient safety concepts.<br><br>**Effective: Yes**<br>**Attrition/Reporting Bias: Low**

			Appraisals for participants - describe patient safety concepts and identifying situations when breakdown in teamwork or communication may contribute to medical error, ability to call effectively on other resources in the system, ability to critically appraise literature and apply evidence-based medicine.		
Nguyen et al 2011	To teach residents about health care reform through debate	Residents at the Department of Physical Medicine and Rehabilitation, Carolinas Medical Center (US)	Debate – policy debate format, cross-examination method, 5 residents assigned to 'affirmative' team and 6 residents to 'opposition' team. Team captains met team and assigned aspects to be researched. Program director was moderator and judge for debate, 2 other judges (chief legal executive and VP of government relations) Follow-up – one month after debate, surveyed residents' attitudes towards health care reform	NA	Debate allowed for complexities in health care delivery to be addressed and encouraged residents to evaluate the larger health care system.<br><br>**Effective: Yes**<br>**Attrition/Reporting Bias: Moderate**
Ong et al 2010	To facilitate learner engagement in a large group with emphasis on clinical application of published evidence through debate	Paediatric surgery trainees at KK Women's and Children's Hospital (Singapore)	Each trainee asked to summarise case, management and relevant articles in 10 minutes using a maximum of five slides, and to allow 2 minutes for rebuttal.<br><br>Debate outcome was judged by an audience vote for the more convincing speaker based on the quoted literature.	Collected feedback using a 5-point Likert scale response on whether the debate met learner needs	87% found it a new and enjoyable style of learning, 75% wanted more of such sessions.<br><br>More than 50% felt that debate helped them to understand the literature better and to apply evidence to actual patients.<br><br>69% felt it helped to understand topic better.<br>**Effective: Yes**<br>**Attrition/Reporting Bias: Low**
Randolph 2007	To evaluate student perceptions about the use of debate	Undergraduate occupational therapy students at	Students given debate assignment one week prior to the debate itself. Each group	Four question survey	Majority of students felt they appreciated better both qualitative and quantitative research methods

		Midwestern University (US)	was assigned an instructor, who provided guidance.<br>Debate: First team presented information for 10 minutes followed by the second team. Each team was then allowed to question the other team for three minutes per question.		(72%), understood the importance of evidence based arguments (71%).<br>87% rated use of debate as a good learning experience and 72% related that debate was a great mechanism for learning.<br><br>**Effective: Yes**<br>**Attrition/Reporting Bias: Low**
Rubin et al 2007	To demonstrate the use of debates as an instructional tool	Undergraduate dental hygiene students at the University of Pittsburgh (US)	Each debate about 20 to 30 mins. Moderated by instructor. Grading – 1) individual presentation, clear, concise thoughtful, researched effort 2) copy of search history 3) overall group presentation	Evaluation of debate took place as small group discussions with faculty, and the attending students also completed a short, eight-item questionnaire immediately following each debate	Student interest in topic heighted and knowledge improved. Debate improved understanding of topic and helped students to organize their thoughts on presented topics<br><br>**Effective: Yes**<br>**Attrition/Reporting Bias: Low**
Shaw 2012	To address controversies in microbiology	Undergraduate sophomore nursing students within an introductory microbiology course at Endicott College (US)	Debates over 1 semester, 30 to 45 mins of class time.<br><br>Procedure – both groups allowed 10 mins to present their points of view<br><br>Post-debate questionnaire to rate value of debate as a learning experience	Post-debate questionnaire: Each student to rate the value of the debate as a learning experience and to disclose whether the student changed his or her opinion on the topic under debate. Each student was assessed for their debate using a grading rubric as well.	97 to 100% of students agreed or strongly agreed that debate improved their understanding of the topic. Correlation between student debate grades and course grades was 83%.<br><br>**Effective: Yes**<br>**Attrition/Reporting Bias: Low**
